# Advances in integrated surgical and rehabilitation treatment for cerebral palsy

**DOI:** 10.3389/fneur.2026.1986453

**Published:** 2026-10-29

**Authors:** Zherui Zhang, Lou Li, Kezhi Li, Chuanxin Yu, Qinchuan Du, Zhiyan Hu, Xiaochao Yu, Jin Zhang, Jun Hu, Yucheng Zhou

**Affiliations:** 1 Department of Orthopedics, Affiliated Calmette Hospital of Kunming Medical University, Kunming, Yunnan, China; 2 Department of Bone and Joint Rehabilitation, North City Hospital of Yun Nan Disabled Rehabilitation Center, Kunming, Yunnan, China; 3 Department of Comprehensive Surgical, Kunming Women and Children's Health Hospital, Kunming, Yunnan, China; 4 Department of Transfusion Medicine, Affiliated Calmette Hospital of Kunming Medical University, Kunming, Yunnan, China

**Keywords:** biomechanics, cerebral palsy, functional reconstruction, integrated therapy, neuroplasticity, rehabilitation, spasticity management, surgical treatment

## Abstract

Cerebral palsy (CP) is a group of motor disorders caused by non-progressive brain injury during development, often accompanied by sensory, cognitive, and behavioral impairments. It remains one of the most common causes of childhood disability. In recent years, the coordinated integration of surgical intervention and rehabilitation has transitioned from a conceptual framework to clinical practice, becoming a core strategy for improving patient function. However, integrated treatment still faces major bottlenecks: insufficient bridging between treatment phases, lack of individualized protocols, and inconsistent outcome measures. This review examines recent advances in integrated surgical and rehabilitative care for CP, covering four core domains: spasticity management (selective dorsal rhizotomy, botulinum toxin injection) with sequential rehabilitation; deformity correction (tendon lengthening, osteotomy) with biomechanically guided individualized rehabilitation; neuromodulation (deep brain stimulation, transcranial magnetic stimulation) with neuroplasticity-targeted training; and emerging technologies including robotic-assisted therapy, artificial intelligence, and digital therapeutics. Surgery creates the anatomical foundation for function; rehabilitation determines the ultimate functional value of surgery. The temporal coordination and strategic synergy between the two are critical for maximizing functional improvement. Future efforts should focus on establishing unified outcome measures for integrated treatment, promoting early intervention combined with regenerative medicine, and leveraging AI to shift from experience-driven to data-driven precision therapy.

## 1 Introduction

Cerebral palsy (CP) is a group of motor disorders caused by non-progressive brain injury during development, often accompanied by sensory, cognitive, and behavioral impairments (1, 2). For decades, CP treatment has been confined to single disciplines, with suboptimal outcomes. Rehabilitation alone can improve joint range of motion and muscle strength to some extent, but cannot reverse the skeletal deformities and joint contractures caused by chronic spasticity and atypical posture (3). Conversely, surgery alone, such as orthopedic procedures or selective dorsal rhizotomy (SDR), can correct deformities or reduce spasticity, but without systematic postoperative rehabilitation, functional gains are often compromised by muscle atrophy, atypical movement patterns, or soft tissue adhesions (4). This dilemma reflects an incomplete understanding of CP pathophysiology: CP is not simply a motor neuron injury but a multi-system syndrome involving muscle, bone, and nerve that evolves over time. Patients vary widely in motor type (spastic, dyskinetic, ataxic), severity (Gross Motor Function Classification System [GMFCS] levels I–V, where level I indicates independent walking and level V indicates severe limitation requiring a wheelchair (5)), and associated sensory, cognitive, and swallowing impairments. No single discipline can address all these dimensions.

Surgery and rehabilitation are complementary, not interchangeable, in CP management. Surgery targets spasticity and structural deformity directly—SDR reduces lower-limb spasticity by partially severing over-excited dorsal root afferents, and orthopedic procedures correct joint contractures and bony deformities through tendon lengthening or osteotomy. However, the success of these procedures depends heavily on systematic postoperative rehabilitation, which leverages neuroplasticity to strengthen residual motor function, consolidate surgical gains, and prevent secondary damage (6, 7). The integrated treatment model emphasizes seamless coordination from preoperative assessment through intraoperative execution to postoperative rehabilitation, aiming to achieve the transition from structural correction to functional reconstruction through multidisciplinary teamwork (8, 9). However, direct evidence that this coordination yields synergistic effects beyond the sum of individual interventions remains limited; most support derives from observational studies and mechanistic reasoning rather than randomized controlled trials. Throughout this review, we distinguish between interventions with established clinical roles (e.g., SDR, orthopedic surgery, conventional physical and occupational therapy) and those that remain experimental or investigational (e.g., deep brain stimulation (DBS) for dystonia in dyskinetic CP, an adjustable neuromodulation technique that delivers electrical pulses to deep brain targets to modulate abnormal neural activity (1, 10), regenerative medicine, AI-driven decision support).

Despite its broad acceptance, the concept of integrated treatment has not yet matured into standardized clinical pathways, evaluation systems, or long-term follow-up mechanisms. Substantial variation exists across centers in preoperative assessment criteria, surgical indications, postoperative rehabilitation protocols, and outcome measures, making cross-study comparisons difficult. Key questions—such as the optimal timing of surgery relative to rehabilitation, the differential treatment responses across CP subtypes, and how to quantify the synergistic effect of combined surgery and rehabilitation—remain without high-quality evidence.

This review aims to: (1) systematically synthesize recent advances in integrated surgical and rehabilitative treatment for CP, covering spasticity management, deformity correction, functional reconstruction, and postoperative rehabilitation; (2) analyze key components of the integrated model, including multidisciplinary team (MDT) construction, perioperative rehabilitation bridging, and individualized protocol design; (3) evaluate the current status and future potential of emerging technologies (robotic therapy, neuromodulation, biomechanical analysis, AI); and (4) identify evidence gaps and future directions to guide clinical practice and research.

Methods: We adopted a narrative approach and did not conduct a systematic review; PRISMA reporting was therefore not applicable. To make the selection process transparent, we used a PRISMA-informed screening workflow. PubMed was searched from inception to 30 August 2026, combining cerebral palsy terms with terms for surgical and rehabilitative interventions (selective dorsal rhizotomy, orthopedic surgery, rehabilitation, neuromodulation, robotic rehabilitation, artificial intelligence). Eligible records were peer-reviewed English-language studies, systematic reviews, meta-analyses, guidelines and expert consensus; conference abstracts, editorials, letters, preprints and non-English publications were excluded. After deduplication, titles and abstracts were screened, followed by full-text assessment; disagreements were resolved by consensus. Evidence was synthesized narratively. We did not perform a formal risk-of-bias assessment or meta-analysis. As only PubMed was searched, studies indexed solely in other databases may have been missed.

## 2 Pathophysiological basis of cerebral palsy and treatment needs

### 2.1 Neural mechanisms and classification of motor disorders

CP pathology involves the cerebral cortex, basal ganglia, cerebellum, and corticospinal tract, resulting in upper motor neuron injury that manifests as spastic, dyskinetic, or ataxic types (2). Spastic CP is the most common subtype (70–80%), caused by corticospinal tract damage that reduces inhibitory input to spinal motor neurons, leading to hyperexcitability and spasticity (11–13). Dyskinetic CP involves basal ganglia-thalamo-cortical loop abnormalities (14), and ataxic CP is primarily associated with cerebellar injury (15). CP classification has direct implications for treatment selection: spastic CP is the primary indication for surgical interventions such as SDR, whereas dyskinetic CP is better suited for neuromodulation (e.g., DBS) combined with rehabilitation. Classification should function as a decision-making tool, not merely a diagnostic label.

### 2.2 Multidimensional impact of functional impairments

Motor impairment limits not only walking and grasping but also profoundly affects daily living activities (16, 17). CP is frequently accompanied by sensory deficits, skeletal deformities, and cognitive impairment, which interact to compound functional burden. Hip dislocation is particularly common in non-ambulatory children and is closely linked to muscle spasticity and strength imbalance (18, 19). Dysphagia affects over 50% of patients, compromising nutritional intake and respiratory safety in severe cases (20). Prolonged immobility leads to joint contractures, muscle atrophy, and osteoporosis, creating a vicious cycle (21–23). These multidimensional impairments do not occur in isolation; they interact through mechanical, metabolic, and inflammatory pathways. Hip dislocation, for example, not only causes pain and limited mobility but also exacerbates scoliosis and respiratory compromise through postural abnormalities, affecting nutritional status and immune function. This underscores the need for a holistic approach in integrated treatment rather than focusing on single functional domains.

### 2.3 Core logic of integrated surgical and rehabilitation treatment

The core logic of integrated treatment is to maximize functional improvement through synergy between surgery and rehabilitation. Surgery (SDR, orthopedic procedures) relieves spasticity and corrects deformity, creating mechanical and anatomical conditions for rehabilitation. Rehabilitation is thought to leverage neuroplasticity to strengthen residual motor function and prevent secondary damage, although direct evidence in CP remains limited (24–26). A key assumption underlying this logic is that structural improvements created by surgery must be captured and utilized by timely rehabilitation, or surgical effects will diminish over time. A time window exists: neuroplasticity is most active in the early postoperative period, and the intensity and quality of rehabilitation during this window directly determine the upper limit of functional recovery. Clinically, this window starts within the first 1 to 2 postoperative days, when passive mobilization and bed-based activities begin after SDR (24), and extends through a critical consolidation phase of 3 to 6 months, during which strengthening and gait training are most effective (27). Beyond this period, gains may plateau, and maintaining rehabilitation adherence becomes the primary challenge. Full synergy from preoperative assessment through postoperative rehabilitation is essential for treatment efficacy (28). However, this time window is often underestimated or overlooked in clinical practice, limiting the translation of surgical gains into functional improvement.

## 3 Preoperative assessment and surgical strategies

### 3.1 Preoperative functional assessment

Surgical decision-making in CP relies heavily on comprehensive preoperative assessment. GMFCS and MACS are internationally accepted core assessment tools; GMFCS level correlates closely with hip dislocation risk (29). The Modified Ashworth Scale (MAS) and surface electromyography (sEMG) quantify spasticity, with sEMG identifying specific overactive muscles contributing to abnormal gait (30). sEMG is typically recorded from lower-limb muscles, commonly showing simplified activation, increased antagonist co-contraction, and altered temporal activation patterns in CP (31, 32). Magnetic resonance imaging (MRI) of the brain and entire neuro-axis identifies brain lesion location and nature and can reveal disqualifying or surgically modifiable abnormalities in SDR candidates (33–35). A major limitation of current assessment systems is their reliance on static measures, lacking quantitative analysis of dynamic functional patterns and compensatory mechanisms. A seemingly simple equinovarus deformity, for instance, may result from ankle joint pathology, hip rotational abnormalities, or trunk compensation—factors that cannot be distinguished by static examination alone. Therefore, three-dimensional gait analysis combined with dynamic sEMG should become standard rather than optional in preoperative evaluation.

### 3.2 Selective dorsal rhizotomy

SDR reduces lower-limb spasticity by partially severing dorsal root afferents, decreasing spinal alpha motor neuron excitability (36, 37). Studies show that SDR significantly improves motor function and quality of life in children with spastic CP, with substantial gains in GMFM-66 scores achieved without sacrificing muscle strength or selective motor control (28, 38). Quantitative syntheses report a pooled mean improvement in GMFM-66 of 3.29 points and a pooled mean reduction in Modified Ashworth Scale scores of −1.98 points (39). These pooled GMFM-66 changes are total-score estimates; domain-level data, usually reported for GMFM-88, show that gains in ambulatory children are concentrated in the standing (D) and walking/running/jumping (E) domains (40). Surgical candidates are typically patients with spastic diplegia or quadriplegia, GMFCS levels I-III, and no severe fixed deformities (26, 41). Systematic postoperative rehabilitation is essential for optimizing gait and consolidating outcomes. Single-level laminectomy has largely replaced traditional multi-level laminectomy, reducing complications such as spinal deformity (18, 36). Intraoperative neurophysiological monitoring enables surgeons to identify and selectively section over-excited nerve rootlets, improving precision and safety. Notably, the long-term outcomes of SDR depend heavily on the quality and continuity of postoperative rehabilitation. Observational comparisons across centers suggest that heterogeneity in functional outcomes may partly reflect differences in rehabilitation protocols, although center-level differences in patient selection, surgical technique, and care delivery cannot be excluded. Randomized trials comparing rehabilitation protocols after SDR remain scarce. The heterogeneity in functional outcomes reported in the literature largely reflects differences in rehabilitation protocols rather than surgical technique itself—cohorts reporting the best outcomes typically implemented structured postoperative rehabilitation programs. This suggests that SDR should be viewed as a treatment package encompassing preoperative selection, intraoperative technique, and postoperative rehabilitation, rather than as a purely surgical procedure. However, high-quality evidence for SDR remains limited: most studies are single-center and retrospective, with few randomized controlled trials. Long-term follow-up data show that some patients reach a functional plateau or even decline 10–15 years post-surgery, warranting cautious evaluation of long-term benefits (38).

### 3.3 Orthopedic surgery: timing and approaches

Orthopedic surgery corrects muscle contractures—defined as permanent shortening of the muscle-tendon unit with loss of elasticity (21)—joint restriction (limited range of motion), and bony deformities caused by spasticity and muscle imbalance (42, 43). Tendon lengthening reduces musculotendinous tension rather than intracompartmental pressure, thereby improving ankle dorsiflexion (44). Tendon lengthening can effectively reduce posterior compartment pressure and improve ankle dorsiflexion. Surgical timing is a critical determinant of long-term outcomes. Because rapid skeletal growth in children with CP may lead to deformity recurrence, there is no universally applicable optimal age for surgery; traditional clinical practice has favored performing corrective procedures before the pubertal growth spurt, but this recommendation is based more on clinical experience than rigorous evidence (45–48). Skeletal maturation rates and deformity progression patterns vary considerably among patients, and surgical timing should be individualized based on bone age assessment, growth curves, and functional deterioration trends. For children with severe spasticity and fixed deformities, combined SDR and orthopedic surgery may be considered—SDR is performed first to reduce spasticity, followed by simultaneous correction of established contractures and bony deformities, addressing both spasticity and deformity in a single treatment pathway (49, 50).

### 3.4 Neuromodulation: deep brain stimulation

In contrast to SDR and orthopedic surgery, which are established procedures, DBS for CP remains experimental. DBS has shown value for choreoathetoid movements and dystonia in dyskinetic CP. The globus pallidus internus (GPi) is the primary target for dystonia treatment, with the cerebellar dentate nucleus and superior cerebellar peduncle emerging as novel targets (51–53). A meta-analysis reported a mean improvement in Burke-Fahn-Marsden Dystonia Rating Scale (BFMDRS) motor scores of 23.6% (54). DBS requires postoperative rehabilitation to optimize outcomes, and its effect on pain relief is particularly notable (25). DBS application in CP remains at an early stage, facing several challenges. First, brain structural abnormalities in CP (e.g., basal ganglia lesions) may affect electrode placement accuracy and efficacy, and existing studies are mostly small case series without large controlled trials. Second, DBS parameter optimization lacks standardized protocols, relying mainly on empirical adjustment, with substantial variation across centers. Third, the interaction between postoperative rehabilitation and DBS stimulation parameters remains poorly understood—for example, whether rehabilitation affects long-term DBS efficacy, and whether DBS parameters should be dynamically adjusted to match rehabilitation phases. Furthermore, indication criteria for DBS vary widely in the literature, from strict restriction to medication-refractory dystonia to extension to mixed movement disorders, making cross-study comparison difficult (25, 53). Closed-loop DBS systems and multimodal electrophysiological-imaging fusion navigation may overcome these limitations, but more high-quality studies are needed to validate their clinical value (Table 1).

**Table 1:** Comparison of major surgical interventions for cerebral palsy.

Procedure	Indications	Core mechanism	Key outcomes	Postoperative rehabilitation	Refs
Selective dorsal rhizotomy (SDR)	Spastic diplegia/quadriplegia, GMFCS I-III	Partially severs dorsal root afferents; reduces spinal alpha motor neuron excitability	GMFM-66 score improves by 3.1 points; MAS significantly reduced; durable spasticity relief	Passive mobilization from day 1–2; progress to standing and gait training	(26, 28, 36–38, 41)
Orthopedic surgery (tendon lengthening/osteotomy)	Fixed joint contractures, bony deformities (equinovarus, hip dislocation, rotational gait)	Corrects biomechanical abnormalities from muscle imbalance; realigns joint axes	Deformity correction; improved gait parameters; prevents deformity progression	Cast immobilization 3–4 weeks; isometric exercises; progressive weight-bearing	(42, 43, 49, 50, 102)
Deep brain stimulation (DBS)	Medication-refractory dyskinetic CP, predominantly dystonia	Modulates abnormal basal ganglia-thalamo-cortical loop activity	BFMDRS motor score improves by ~30%; notable pain relief	Postoperative parameter optimization; combined with rehabilitation training	(25, 51–53)
Intrathecal baclofen pump (ITB)	Severe spasticity, GMFCS IV-V, oral medication failure	Continuous intrathecal baclofen infusion; inhibits spinal-level excitatory transmission	Effective reduction of generalized spasticity; improved ease of care	Regular pump parameter adjustment; monitor for complications	(118–120)
Selective peripheral neurectomy (SPN)	Focal spasticity (e.g., obturator nerve, tibial nerve branches)	Selectively severs over-excited peripheral nerve branches	Localized reduction in muscle tone; improved joint range of motion	Short-term immobilization; progressive strength and functional training	(25, 121)

## 4 Individualized rehabilitation: approaches and advances

### 4.1 Physical therapy: foundations and optimization

Physical therapy is the foundation of rehabilitation for children with cerebral palsy, including task-oriented training, high-intensity repetitive practice, progressive resistance training, balance training, and gait training. The duration and dose of physical therapy should be individualized rather than fixed. No universal minimum effective dose has been established, and a Bayesian network dose–response meta-analysis confirms that exercise dose and motor skill improvement follow a nonlinear relationship (55). Dosing should be adjusted according to GMFCS level, age, motor phenotype, and comorbidities. Higher therapy intensity generally yields superior functional outcomes, though optimal dosing remains undefined (56). The therapeutic aim is to induce motor cortex reorganization through repetitive task practice, consolidate postoperative correction, and prevent muscle atrophy and contracture recurrence. Outcomes should be monitored with GMFM-66, gait parameters, and muscle strength (57, 58).

### 4.2 Occupational therapy and activities of daily living

Occupational therapy focuses on improving independence and participation in daily living activities through upper-limb function training, including dressing and feeding (59). Constraint-induced movement therapy (CIMT) is a key intervention for hemiplegic CP (60–62). The key to occupational therapy is not the technology itself, but the alignment of training tasks with the child’s real-life scenarios and interests. Gamified hand function training, for example, engages active participation more effectively than repetitive equipment-based exercises, and active engagement is considered a key facilitator of use-dependent neuroplasticity, although its causal contribution in CP remains to be defined.

### 4.3 Speech and swallowing rehabilitation

Speech impairment affects up to 89% of children with CP, and dysphagia is also common (20). Oral motor training, respiratory control, and vocal exercises are core interventions. For severe dysphagia, adjusting feeding posture and food consistency can reduce aspiration risk (63). Telerehabilitation provides a new avenue for speech therapy (64). Speech and swallowing function are often overlooked in discussions of integrated treatment. Dysphagia directly affects nutritional status and respiratory safety, and is a major determinant of overall prognosis. It should be included in routine integrated treatment assessment and management rather than treated as an independent issue.

### 4.4 Innovative rehabilitation technologies

Some of these technologies, such as functional electrical stimulation for foot drop, are already in clinical use, whereas others, including robotic exoskeletons and smart wearables, remain investigational or are still being optimized. Robotic-assisted rehabilitation provides high-intensity, reproducible, and precise training. Lower-limb exoskeletons and upper-limb rehabilitation robots are widely used in CP rehabilitation (65, 66). Meta-analyses of randomized controlled trials indicate that robotic-assisted gait training significantly improves motor function (SMD = 0.89) and gait (SMD = 0.62) in children with cerebral palsy (67). Equinus is the most common deformity in CP, and gastrocsoleus lengthening (GSL) is the most common surgical intervention for this deformity (68). The typical gait deviation in CP is true or apparent equinus, caused by shortening and/or spasticity of the gastrocnemius and soleus, which restricts ankle dorsiflexion rather than isolated dorsiflexor weakness. Foot drop, defined as persistent ankle plantarflexion during swing, is uncommon as a primary presentation in CP and most often occurs after gastrocsoleus lengthening, with a higher incidence in unilateral than bilateral CP (69). FES should therefore be described as targeting insufficient swing-phase dorsiflexion or dynamic equinus, not true foot drop. Smart wearable devices facilitate home-based rehabilitation and remote monitoring (70, 71). A common advantage of these technologies is their ability to quantify training parameters and monitor movement performance in real time, providing a data foundation for objective outcome assessment and dynamic protocol adjustment. However, high equipment costs, operational complexity, and limited home accessibility constrain widespread adoption. Developing low-cost, user-friendly, home-compatible rehabilitation devices is a key direction for technology dissemination (Table 2).

**Table 2:** Rehabilitation approaches for cerebral palsy and their synergistic mechanisms with surgery.

Type	Core methods	Neuroplasticity mechanism	Synergy with surgery	Key outcome measures	Refs
Physical therapy	Task-oriented training, high-intensity repetitive training, progressive resistance training	Induces motor cortex reorganization through repetitive task practice	Consolidates postoperative correction; prevents muscle atrophy and contracture recurrence	GMFM-66 score, gait parameters, muscle strength	(57, 58, 122–124)
Occupational therapy	CIMT, bimanual intensive training, ADL training, VR-assisted training	Restrains unaffected side; forces functional reorganization of affected side	Postoperative upper-limb functional reconstruction; converts structural gains into function	MACS level, House score, ADL independence	(45, 59, 125–127)
Speech and swallowing therapy	Oral motor training, respiratory control, compensatory swallowing strategies, augmentative communication devices	Functional reorganization of orofacial motor cortex	Improves postoperative nutritional status; prevents aspiration pneumonia	Speech clarity, SWAL-QOL, aspiration rate	(20, 63, 64)
Robotic-assisted rehabilitation	Lower-limb exoskeleton (Lokomat), upper-limb rehabilitation robots, portable ankle robots	Provides high-intensity, reproducible, task-oriented training; promotes motor learning	Early postoperative intervention; compensates for insufficient traditional rehabilitation intensity	Joint range of motion, walking speed, 6-min walk test	(65, 66, 70, 71)
Neuromodulation + rehabilitation	rTMS/tDCS priming followed by rehabilitation; spinal cord stimulation (SCS)	Modulates cortical excitability; opens a ‘time window’ for motor learning	Rehabilitation during the cortical plasticity window generates synergistic effects	MAS score, motor evoked potentials, gait symmetry	(96–99)

## 5 Perioperative rehabilitation bridging in integrated treatment

### 5.1 Preoperative rehabilitation: optimizing patient readiness

Preoperative rehabilitation (prehabilitation) aims to optimize patient status through strength training, joint range-of-motion maintenance, and cardiopulmonary conditioning. Strength training of core and lower-limb muscles provides the foundation for early postoperative mobilization. Range-of-motion training through passive or active-assisted stretching prevents joint contractures caused by chronic spasticity. Preoperative botulinum toxin injection can temporarily reduce localized muscle spasticity, providing a more accurate assessment window to distinguish the relative contributions of spasticity versus fixed contracture to deformity (27, 72). Psychological support interventions can alleviate anxiety in children and families, establish realistic expectations for the surgical and rehabilitation process, and improve postoperative rehabilitation adherence (49). Preoperative rehabilitation is often undervalued, but its importance rivals that of postoperative rehabilitation. Preoperative strength reserve and joint mobility directly determine the starting point and pace of postoperative recovery, while psychological preparation influences the family’s long-term commitment to rehabilitation. Incorporating prehabilitation into the standard integrated treatment protocol is a low-cost, high-yield strategy for improving overall outcomes. Prehabilitation should currently be regarded as an emerging strategy with limited direct evidence, rather than an established standard of care.

### 5.2 Early postoperative rehabilitation

The core goals of early postoperative rehabilitation are to control the acute response, protect the surgical site, prevent complications, and promote early functional recovery. After SDR, passive bed-based activities can begin on postoperative days 1–2, progressing to sitting and standing training, with orthoses to maintain functional joint positions and counteract spasticity recurrence and deformity (26). After orthopedic surgery, rehabilitation protocols should be individualized based on the procedure. Most orthopedic surgeries require cast immobilization for 3–4 weeks to ensure bone healing, during which the focus is on isometric exercises and active/passive mobilization of adjacent joints. Early progressive weight-bearing should be encouraged once fixation stability is confirmed, to avoid the functional decline and bone loss associated with prolonged immobilization (28). Postoperative rehabilitation should not be viewed as a mere appendage to surgery; it is a necessary component of surgical success. Evidence supporting this claim is largely indirect: early mobilization after SDR is recommended and observational data suggest it may facilitate functional recovery (26), whereas insufficient or delayed rehabilitation may be associated with poorer functional recovery and secondary complications; randomized evidence for specific timing and intensity is scarce.

### 5.3 Consolidation phase: long-term management

The 3–6 month period after surgery is a critical window for functional recovery, with emphasis on strengthening and gait training to correct abnormal movement patterns (73). Long-term management relies on regular follow-up assessment and dynamic adjustment of rehabilitation plans, progressing from physical therapy to functional occupational therapy and community activity participation (74, 75). The greatest challenge in consolidation-phase rehabilitation is not technical but adherence. Over time, family commitment to rehabilitation often declines as functional gains plateau. Establishing phased, quantifiable short-term goals and demonstrating progress through regular assessments are effective strategies for maintaining long-term rehabilitation motivation (Figure 1).

**Figure 1:**
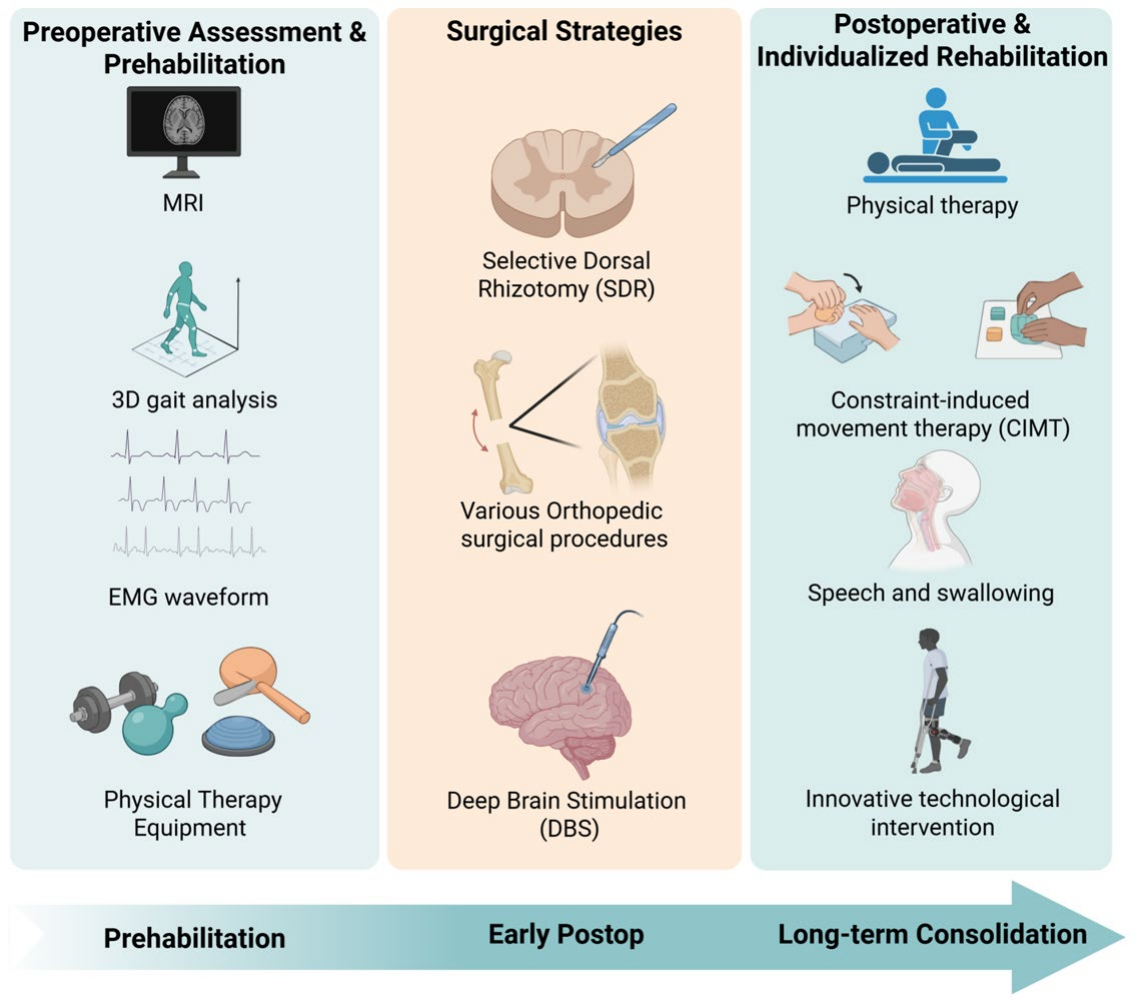
Integrated perioperative pathway for cerebral palsy: aligning surgical strategies with rehabilitation timing. This three-phase pathway aligns preoperative assessment/prehabilitation, surgical strategies (SDR, orthopedics, DBS), and postoperative rehabilitation (PT, CIMT, speech/tech) along a temporal axis, emphasizing surgery-rehabilitation synergy for optimal functional recovery.

## 6 Multidisciplinary collaboration and integrated treatment practice

### 6.1 Building the multidisciplinary team

The MDT requires participation from neurosurgery, orthopedic surgery, rehabilitation medicine, physical therapists, occupational therapists, speech therapists, and psychologists (76–79). Neurosurgeons and orthopedic surgeons assess and perform necessary surgical interventions; rehabilitation physicians lead the development of long-term rehabilitation plans; physical therapists focus on improving gross motor function; occupational therapists work on enhancing activities of daily living. Regular MDT meetings ensure adequate information sharing and treatment decision consistency, preventing treatment conflicts or delays caused by information silos (80). Establishing standardized treatment pathways is key to implementing and continuously improving MDT, with clear specifications for diagnosis, assessment, treatment, and follow-up (81). However, MDT implementation often remains superficial due to high communication costs, unclear role definitions, and uneven resource allocation. An effective MDT requires a clear coordinator (typically a rehabilitation physician) to integrate multidisciplinary input and establish a patient-centered decision framework rather than a specialty-centered consultation model. MDT is widely recommended but remains evidence-limited; it should be distinguished from established single-specialty interventions. Notably, the evidence for MDT effectiveness in CP treatment is largely descriptive, with few high-quality studies quantifying the advantages of MDT over traditional single-specialty care. The costs, communication efficiency, and specific impact of MDT on patient outcomes require rigorous controlled studies.

### 6.2 Integrating family and community support

Training family members to perform rehabilitation exercises is a key strategy for improving treatment adherence, integrating rehabilitation into daily routines (82, 83). Through systematic family training, parents can learn basic rehabilitation techniques—using a standing frame for weight-bearing exercises or gamified balance training—significantly increasing treatment frequency and effectiveness. Community resources (special education schools, community rehabilitation centers) provide structured and inclusive environments for children to develop social skills and build confidence through group activities. Telerehabilitation platforms overcome geographical barriers, allowing therapists to observe training movements and correct errors in real time via video conferencing, enabling the sharing of high-quality rehabilitation resources (84, 85). A family-community-hospital integrated support network is the foundation for maintaining long-term rehabilitation outcomes. The family is the primary agent of rehabilitation, the community is its extension, and the hospital serves as the guiding center—all three are indispensable.

### 6.3 Outcome measures for integrated treatment

GMFM-66 is the internationally accepted gold standard for motor function assessment (86). The GMFM-88 provides dimension-level scores and is applicable to a broader pediatric population, whereas the GMFM-66 is CP-specific and interval-level; the GMFM-66 is more responsive to change (87, 88). Three-dimensional gait analysis and EMG provide objective quantitative measures. Functional walking tests widely used in CP include the 10-meter walk test (10mWT) for walking speed, the 6-min walk test (6MWT) for endurance, and the Timed Up and Go (TUG) test for functional mobility (89–91). The CPQoL-Child includes child self-report and caregiver-proxy versions; given their limited agreement, both should be considered for comprehensive assessment (92). However, heterogeneity in outcome measures across studies limits comparability. To address this, an international consensus has recommended seven core outcome measures for ambulant children with CP after lower limb orthopedic surgery, covering eight domains including pain and fatigue, lower limb structure, motor function, daily life activities, gait-related outcomes, physical activity, independence, and quality of life (93) (Table 3).

**Table 3:** Illustrative integrated treatment considerations by cerebral palsy subtype.

CP subtype	GMFCS level	Core impairment	Preferred surgical intervention	Rehabilitation focus	Illustrative integrated pathway	Refs
Spastic diplegia	I-III	Lower-limb spasticity; hip adduction, knee flexion, equinovarus	SDR as the primary approach, combined with multi-level orthopedic surgery	Postoperative gait training, strength training, balance training	Individualized sequence may include assessment, SDR (if indicated), early postoperative rehabilitation, orthopedic surgery as needed, and long-term training.	(18, 26, 28, 36–38, 41–43, 49, 50, 102)
Spastic quadriplegia	IV-V	Severe spasticity in all limbs; often accompanied by hip dislocation, scoliosis	ITB pump implantation, hip reconstruction, spinal fusion	Passive range of motion maintenance, positioning, assistive devices	Management may involve ITB for spasticity control, with orthopedic surgery and rehabilitative care as appropriate, and long-term follow-up tailored to patient needs.	(18, 50, 128)
Dyskinetic	I-IV	Dystonia, choreoathetosis; often involves orofacial region	DBS (GPi or cerebellar dentate nucleus target)	Posture control, coordination training, medication management	DBS evaluation and possible implantation may be considered, integrated with individualized rehabilitation.	(25, 51–53)
Hemiplegic	I-II	Unilateral spasticity; upper-limb function significantly limited	Upper-limb orthopedic surgery (tendon transfer, joint fusion), SPN	CIMT, bimanual training, mirror therapy	Functional assessment (SHUEE) → SPN/CIMT→Orthopedic surgery→Intensive occupational therapy	(42, 59, 125–127)
Mixed	I-V	Coexisting spasticity and dyskinesia; most challenging to treat	Multi-modal combination (DBS + orthopedic, ITB + orthopedic)	Comprehensive rehabilitation, multidisciplinary management	MDT assessment→Identify primary problem→Prioritize intervention→Dynamic adjustment	(76, 77, 81)

The pathways shown are illustrative examples; clinical decisions should be highly individualized based on patient-specific factors, and the sequence of interventions may vary.

## 7 Emerging technologies and integrated treatment

### 7.1 Robotic-assisted integrated treatment

Surgical robots use high-precision navigation to significantly improve the accuracy of pedicle screw placement, reduce the risk of neurovascular injury from human positioning error, and decrease surgical trauma and blood loss, creating favorable conditions for early postoperative rehabilitation (94). In GMFCS IV–V patients with severe postural deformity, pedicle screw fixation primarily aims to correct spinal deformity, rather than restore ambulation. A meta-analysis of 7 studies (584 patients) showed that robot-assisted pedicle screw placement in scoliosis surgery significantly improved clinically acceptable screw placement rates compared with freehand technique (95). However, evidence for its use and effectiveness in GMFCS IV–V CP patients with neuromuscular scoliosis remains limited. Rehabilitation robots provide high-intensity, reproducible, task-oriented training, compensating for the limitations of traditional manual therapy in intensity and quantitative assessment. Robotic-assisted gait training (RAGT) effectively improves hip range of motion, limb symmetry, and propulsion (70). Portable rehabilitation robots combining smart-controlled passive stretching with game-based active movement training significantly improve joint range of motion, strength, and selective motor control. The true value of robotic technology lies not in replacing therapists but in providing precision measurement and individualized modulation that traditional methods cannot achieve. Future data exchange between surgical and rehabilitation robots could create a fully digital closed loop from surgical planning to rehabilitation training—intraoperative biomechanical data recorded by the surgical robot directly guiding the rehabilitation robot to develop individualized training protocols—representing true integration.

### 7.2 Synergy between neuromodulation and rehabilitation

Transcranial magnetic stimulation (TMS) and transcranial direct current stimulation (tDCS) can modulate cortical excitability and are being explored as adjunctive rehabilitation tools (96, 97). Emerging evidence suggests that applying TMS or tDCS before or during rehabilitation may enhance motor cortex plasticity and increase responsiveness to subsequent training, but effects are heterogeneous and optimal protocols remain undefined (98, 99). Spinal cord stimulation (SCS) sends electrical pulses to the dorsal columns, modulating spinal reflexes and motor neuron excitability, and has shown preliminary potential to reduce lower-limb muscle tone and improve gait in selected patients (100). Closed-loop neuromodulation systems adjust stimulation parameters in real time based on physiological feedback (e.g., local field potentials or movement state), a promising strategy for more precise individualized treatment, particularly for fluctuating symptoms such as dyskinesia or paroxysmal spasticity, but clinical validation is still limited (101). A proposed synergy between neuromodulation and rehabilitation may require precise temporal matching, for example, rehabilitation during a TMS-induced period of heightened cortical excitability might yield better outcomes than non-synchronized treatment; however, evidence remains scarce. However, high-quality evidence on optimal stimulation parameters, timing windows, and training content combinations remains scarce, and this should be a priority for future research.

### 7.3 Biomechanical analysis and individualized treatment

Preoperative gait analysis integrates kinematic, kinetic, and dynamic EMG data to objectively identify abnormal gait patterns (42). Computer simulation of surgical outcomes can predict the effect of specific procedures on postoperative gait function (43). Postoperative biomechanical assessment validates surgical effects and enables dynamic adjustment of rehabilitation intensity (102, 103). A systematic review of gait parameters in children with bilateral spastic CP found that spatiotemporal and kinematic parameters, particularly joint angles in the sagittal plane, are reported most frequently, whereas kinetic and surface EMG data are used less often (104). Standardization remains insufficient, with pelvic and hip rotation and step length showing the greatest variability across centers (105). Biomechanical analysis is transitioning from a research tool to a routine clinical method, but still faces challenges including insufficient standardization of data interpretation, high equipment costs, and shortage of trained personnel. Transforming biomechanical analysis from a tool exclusive to large research centers into a clinical decision-making aid accessible to hospitals at all levels is key to advancing precision in integrated treatment.

## 8 Challenges and future directions

### 8.1 Resource allocation and accessibility

CP prevalence is particularly high in low- and middle-income countries, yet these regions lack adequate medical infrastructure and rehabilitation resources (106, 107). There is a global shortage of rehabilitation therapists, and their level of expertise directly affects treatment quality. In resource-limited areas, therapists often lack specialized training in CP, making it difficult to deliver high-quality rehabilitation services (108). Government policy support, particularly health insurance coverage and rehabilitation subsidies, is a decisive factor in improving treatment accessibility. Expanding insurance coverage, providing rehabilitation subsidies, and establishing dedicated screening and follow-up programs can significantly improve access to integrated treatment for CP patients (85, 109). In resource-constrained settings, prioritizing low-cost, high-impact interventions (such as family training and telerehabilitation) and establishing a tiered referral system based on severity stratification may be more pragmatic than pursuing high-end technologies.

### 8.2 Individual patient variability and treatment matching

CP is highly heterogeneous in clinical presentation, and CP type, severity, and age significantly influence treatment response (28). Children with spastic diplegia show greater motor function improvement after SDR than those with spastic quadriplegia. Patients younger than 10 years have a higher rate of GMFCS level improvement after surgery compared with those older than 10 years, suggesting an optimal treatment window. Cognitive impairment or behavioral problems can significantly affect postoperative rehabilitation adherence, requiring customized intervention strategies including simplified instructions, visual cues, gamified training, and increased family involvement (41). Genetic factors and comorbidities (epilepsy, intellectual disability) increase treatment complexity and require multidisciplinary comprehensive assessment (2). True integrated treatment does not apply a uniform protocol to all patients but develops differentiated strategies based on individual characteristics (CP type, severity, age, cognitive level, family support). Machine learning-based patient stratification models may help clinicians achieve precise treatment matching in the future.

### 8.3 Long-term follow-up and efficacy validation

A major limitation of the current evidence base is the scarcity of long-term outcome data; most cited studies report relatively short follow-up periods, and the durability of functional gains into adulthood remains largely unknown. The long-term efficacy of SDR combined with rehabilitation has been validated in multiple studies, with functional improvements maintained for 10–15 years post-surgery (25, 28). However, the lack of unified outcome standards remains a pressing issue (86). Big data and AI technologies offer new opportunities for identifying optimal treatment patterns (110). We call for the establishment of national or international CP integrated treatment registries, through standardized data collection and long-term follow-up, to accumulate high-quality real-world evidence that can inform treatment optimization and health economic evaluation.

## 9 International frontiers and clinical practice trends

### 9.1 Integrating early intervention with integrated treatment

Early diagnosis in infancy is the prerequisite for initiating intervention. Combining neuroimaging with general movement assessment significantly improves the accuracy of CP prediction (111, 112). Early intervention capitalizes on the peak window of infant neuroplasticity to avert secondary deformities. Compared with those who receive early intervention, children with CP who do not are at substantially increased risk of developing joint contractures (113, 114). Integrating early intervention with the integrated treatment model—initiating multidisciplinary management from infancy—could achieve full-cycle coverage from prevention to treatment. Moving the starting point of integrated treatment to infancy, realizing a paradigm shift from waiting for treatment to active prevention, may be one of the most transformative directions in CP management. Although early intervention itself is established, its integration with the full integrated treatment model remains exploratory.

### 9.2 Regenerative medicine and integrated treatment

Preliminary randomized evidence suggests that mesenchymal stem cell transplantation combined with rehabilitation may improve gross motor function in some children with CP (115, 116), but this approach remains investigational. Growth factor therapy combined with rehabilitation training has been proposed to produce synergistic effects, but clinical evidence remains limited. Tissue engineering provides new materials and methods for orthopedic surgery. However, the combination of regenerative medicine and integrated treatment remains at the proof-of-concept stage, facing key challenges including cell source standardization, long-term safety evaluation, and optimal timing matching with rehabilitation training. If future stem cell or tissue-engineering therapies can partially repair neural damage or improve musculoskeletal structure, rehabilitation could potentially move beyond functional compensation toward neural reconstruction, but this remains a long-term hypothesis rather than an established clinical reality.

### 9.3 Artificial intelligence and digital therapeutics

Machine learning algorithms can efficiently analyze gait and EMG data, providing objective evidence for clinical decision-making (103, 110). Deep learning models can accurately estimate kinetic parameters from kinematic data, replacing traditional force-plate methods (117). Digital therapeutics improve treatment adherence through mobile applications and gamified design (70). Wearable sensors combined with deep learning enable 24-h real-world movement behavior monitoring (102). AI technologies not only improve the precision of diagnosis and assessment but also provide technical support for telerehabilitation and dynamic adjustment of individualized treatment plans (Table 4). We predict that the impact of AI on CP integrated treatment will manifest at three levels: decision support (identifying optimal treatment patterns from data), enabling rehabilitation (automated generation and dynamic adjustment of individualized training protocols through intelligent algorithms), and connecting the care pathway (bridging data silos between preoperative assessment, surgical planning, rehabilitation training, and follow-up monitoring), ultimately achieving truly intelligent integrated treatment (Figure 2).

**Table 4:** Evidence hierarchy for integrated surgical and rehabilitative interventions in cerebral palsy.

Intervention	Evidence level	Representative evidence	Interpretation	Key limitations
Botulinum toxin A	A–B	Multiple RCT meta-analyses; moderate evidence for limited functional benefit (27, 72)	Recommended for spasticity reduction	Limited functional gain; short-term effects
Strength/resistance training	A–B	Meta-analyses of RCTs; improves strength with moderate certainty (57, 58)	Recommended for strength	Functional translation uncertain
CIMT	A–B	Cochrane review; multiple RCTs and meta-analyses (60–62)	Recommended	GRADE certainty very low; publication bias detected
SDR	A–B	Pooled meta-analysis of RCTs; clinical importance not established (28, 38)	Conditionally recommended	Small samples; MCIB not met; long-term safety unclear
ITB	B–C	Systematic review/meta-analysis; effective for spasticity but low evidence level (120)	Conditionally recommended	Low certainty; pump complications
Orthopedic surgery (SEMLS)	C	Systematic reviews of observational studies; gait improvement reported (42, 43, 45–50)	Conditionally recommended	Timing not tested in RCTs; evidence limited to observational data
Robotic/wearable rehabilitation	A–B	Meta-analysis of multiple RCTs; combined RAGT+CGT superior (65, 66, 70, 71)	Promising	Moderate-to-high heterogeneity
TMS/tDCS/SCS	B–C	Systematic reviews and RCTs; no significant upper-limb effect for rTMS/tDCS (96–100)	Exploratory	Substantial heterogeneity; optimal parameters unknown
Regenerative medicine	B	Systematic review/meta-analysis of RCTs; safe and effective (115, 116)	Experimental	Standardized protocols needed
DBS	C	Systematic reviews; GPi DBS may improve motor symptoms (51–53)	Exploratory	Very low quality evidence; no large trials
SPN	C	Reviews; case series; limited high-quality studies (25, 121)	Experience-based	Limited controlled data
Prehabilitation	C	Case series; clinical experience (49)	Exploratory	No RCTs
MDT	C	Descriptive studies; guideline-based (76–81)	Experience-based	No controlled comparisons

Evidence levels were assigned by the authors based on study design, number, consistency, and risk of bias of the available evidence: A, multiple RCTs or meta-analyses with consistent findings and at least moderate certainty; B, single or small RCTs, RCTs with important limitations, or meta-analyses with low certainty; C, mainly observational studies, case series, or mechanistic reasoning. These are qualitative judgements for a narrative review and do not represent formal GRADE assessments. GRADE certainty ratings from cited meta-analyses are noted where available. MCIB, minimal clinically important benefit; PRT, progressive resistance training; SEMLS, single-event multilevel surgery; RAGT, robotic-assisted gait training; CGT, conventional gait training; NIBS, non-invasive brain stimulation; SCS, spinal cord stimulation; SPN, selective peripheral neurotomy.

**Figure 2:**
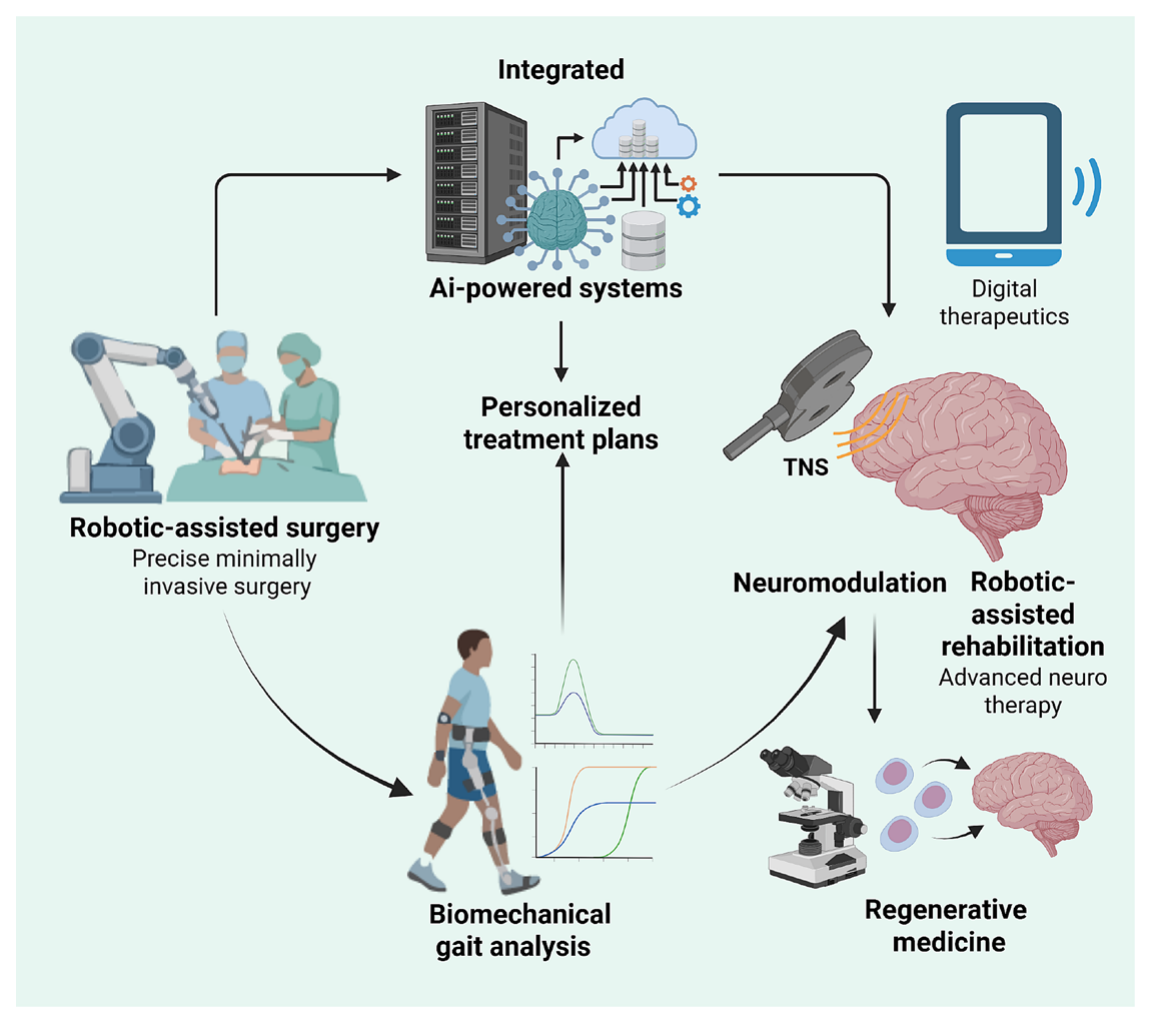
A closed-loop intelligent framework for cerebral palsy: integrating technology, data, and future paradigms. This framework integrates AI, robotic surgery/rehabilitation, neuromodulation, gait analysis, and regenerative medicine into a dynamic system that enables data-driven, personalized treatment, shifting from experience-based to precision care.

## 10 Conclusion

Integrated treatment represents a fundamental shift in the CP management paradigm, from single-discipline intervention to multi-system coordinated integration. A key theme emerging from this review is the need to distinguish established interventions from those still under investigation. The evidence reviewed here reveals a core paradox: although mechanistic studies and observational cohorts support a synergistic effect of surgery and rehabilitation, direct randomized evidence demonstrating that combined treatment exceeds the sum of individual effects is sparse. This recognition has not translated into standardized, generalizable clinical pathways.

Standardized functional tests, such as GMFM-66, three-dimensional gait analysis, the 10-meter walk test, the 6-min walk test, the Timed Up and Go test, and the CPQoL-Child, are widely reported, and specific improvements in these outcomes following various interventions have been documented. However, no dedicated instrument exists to quantify the synergistic effect of combined surgery and rehabilitation; existing measures capture single-dimension changes and cannot capture the interaction between the two modalities.

The most prominent gaps in the current literature cluster around three areas. First, in outcome measurement: no dedicated instrument exists to quantify the synergistic effect of combined surgery and rehabilitation; existing measures (GMFM-66, gait parameters) only capture single-dimension changes and cannot capture the interaction between the two modalities, severely limiting optimization and comparison of integrated treatment protocols. Second, in patient stratification: clinical decisions still rely heavily on coarse indicators such as GMFCS level, lacking precision classification models based on multimodal data (imaging, electrophysiology, biomechanics, genomics), leading to poor matching between treatment protocols and individual patient characteristics. Third, in evidence quality: most studies have follow-up periods shorter than 5 years, small sample sizes, and lack multi-center validation, leaving the core prognostic question unanswered, whether functional improvements are sustained into adulthood.

Looking ahead, three directions hold the greatest transformative potential. First, the deep integration of early intervention with integrated treatment, shifting the starting point to the infant peak of neuroplasticity and leveraging the intervention window of neurodevelopment, to achieve a paradigm shift from passive treatment to active prevention. Second, the integration of regenerative medicine: if stem cell therapy or tissue engineering can partially repair neural damage or improve musculoskeletal structure, rehabilitation would upgrade from functional compensation to true neural reconstruction, redefining the biological foundation of rehabilitation. Third, AI-driven decision support systems: by integrating multimodal clinical data, these systems can enable the transition from experience-based to data-driven decision-making, making “the right treatment combination for the right patient at the right time” an achievable reality. Achieving this transformation will require coordinated efforts from academia, clinical practice, and industry, and a shift in research design from short-term efficacy validation to long-term value creation.

## Glossary

ADL: Activities of daily living
AI: Artificial intelligence
BoNT-A: Botulinum toxin type A
CIMT: Constraint-induced movement therapy
CP: Cerebral palsy
CPQOL: Cerebral Palsy Quality of Life
DBS: Deep brain stimulation
EMG: Electromyography
GMFCS: Gross Motor Function Classification System
GMFM-66: Gross Motor Function Measure-66
GPi: Globus pallidus internus
MACS: Manual Ability Classification System
MAS: Modified Ashworth Scale
MDT: Multidisciplinary team
MRI: Magnetic resonance imaging
RAGT: Robotic-assisted gait training
SCS: Spinal cord stimulation
SDR: Selective dorsal rhizotomy
tDCS: Transcranial direct current stimulation
TMS: Transcranial magnetic stimulation
